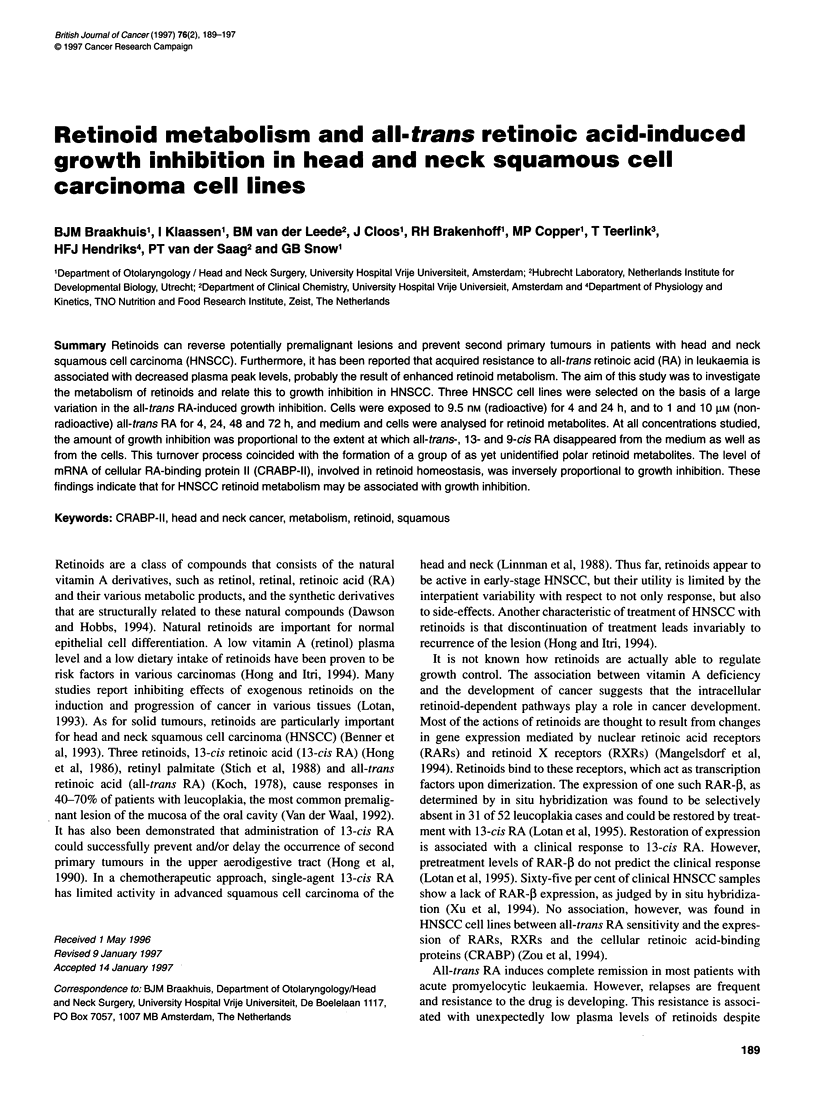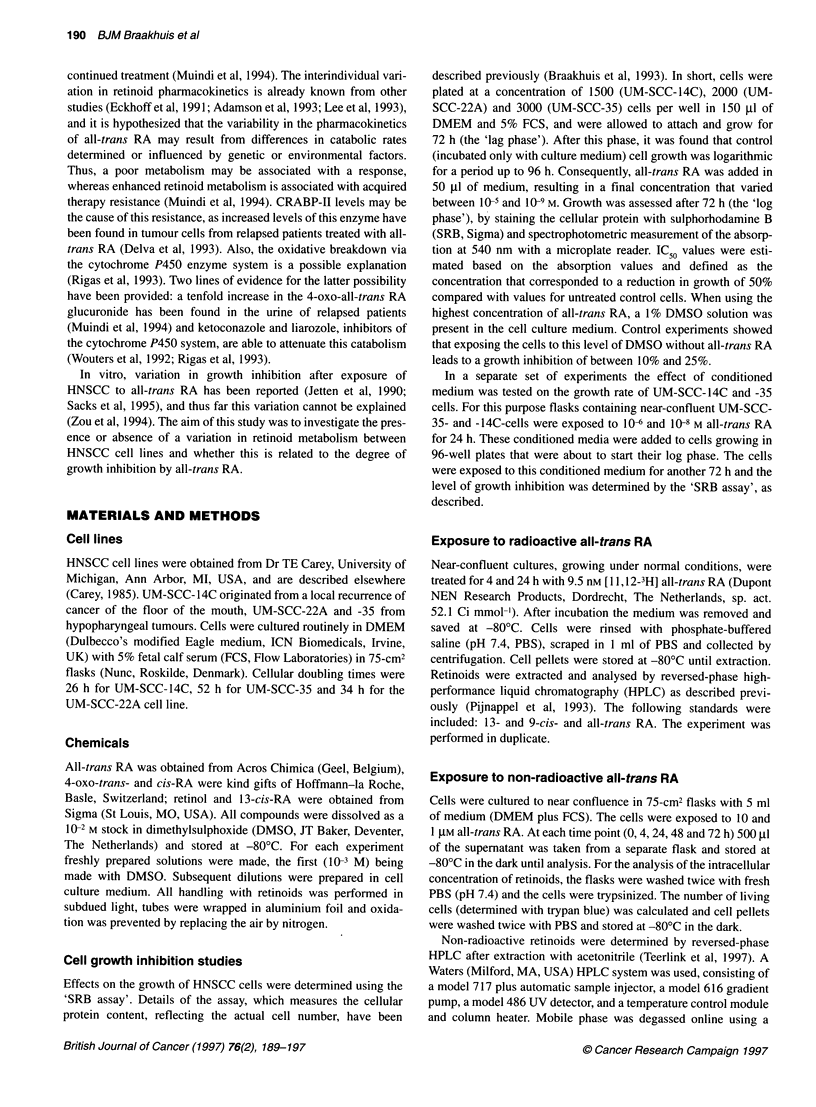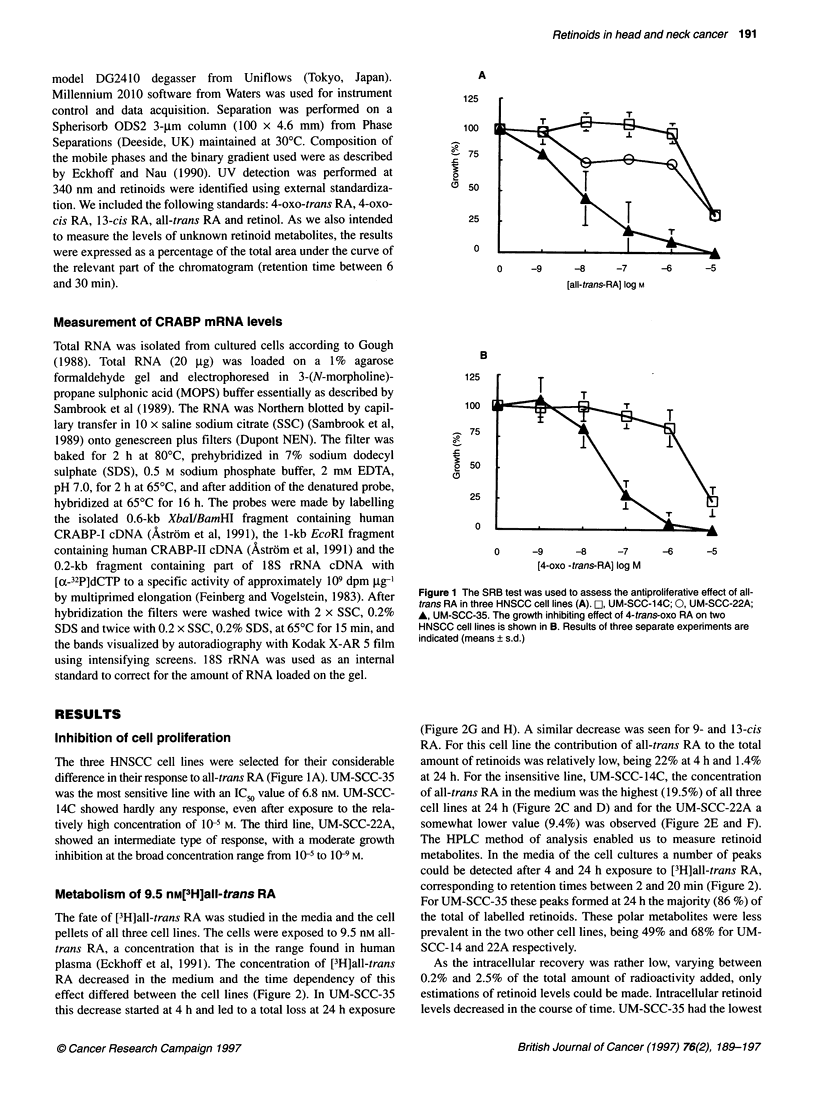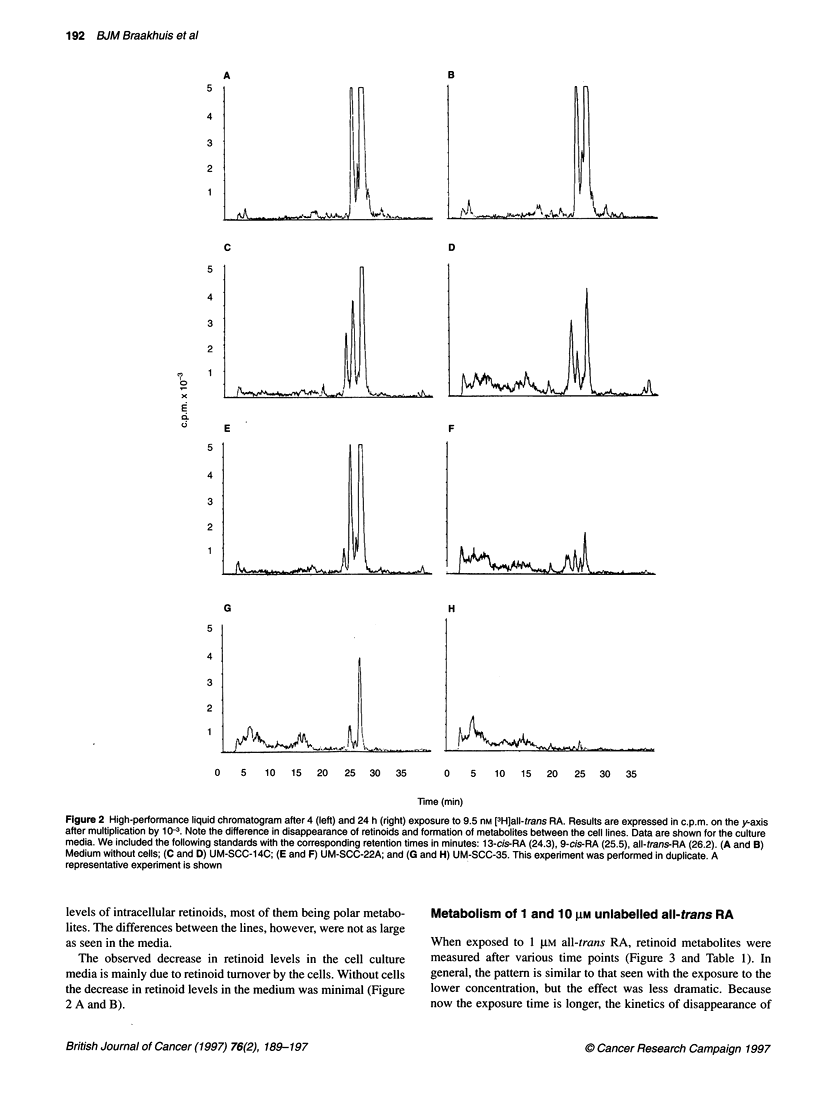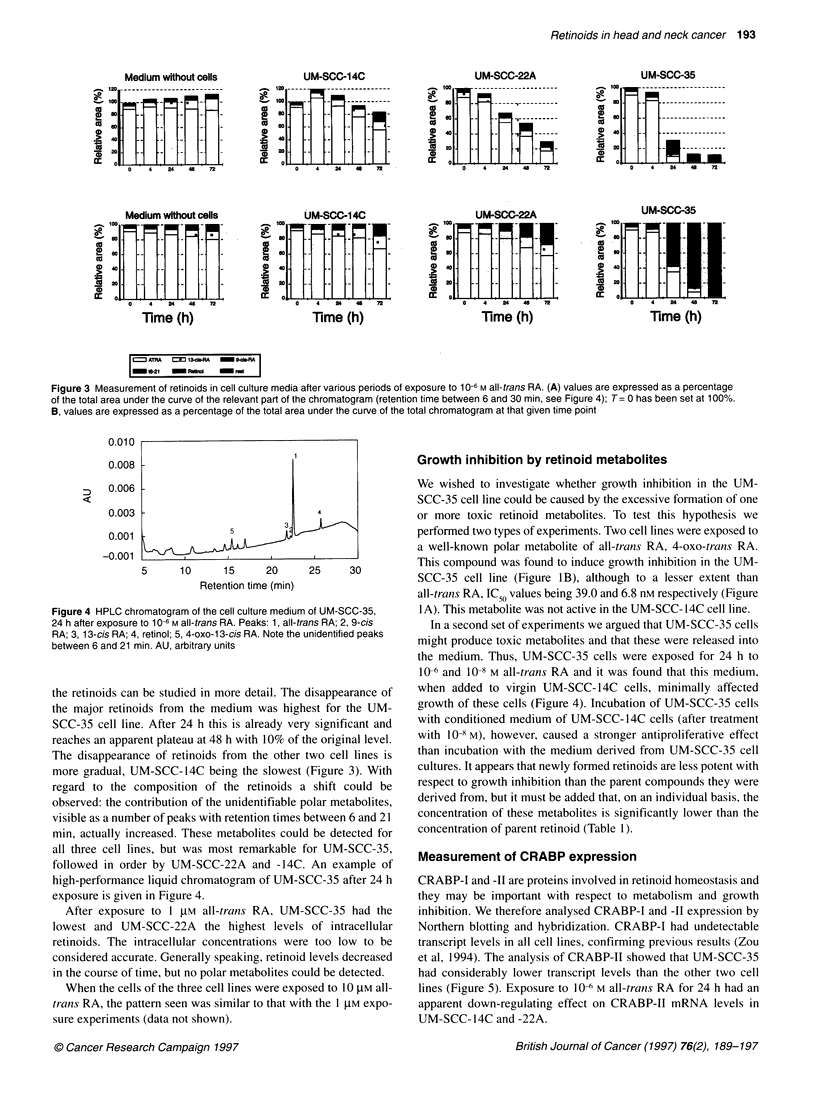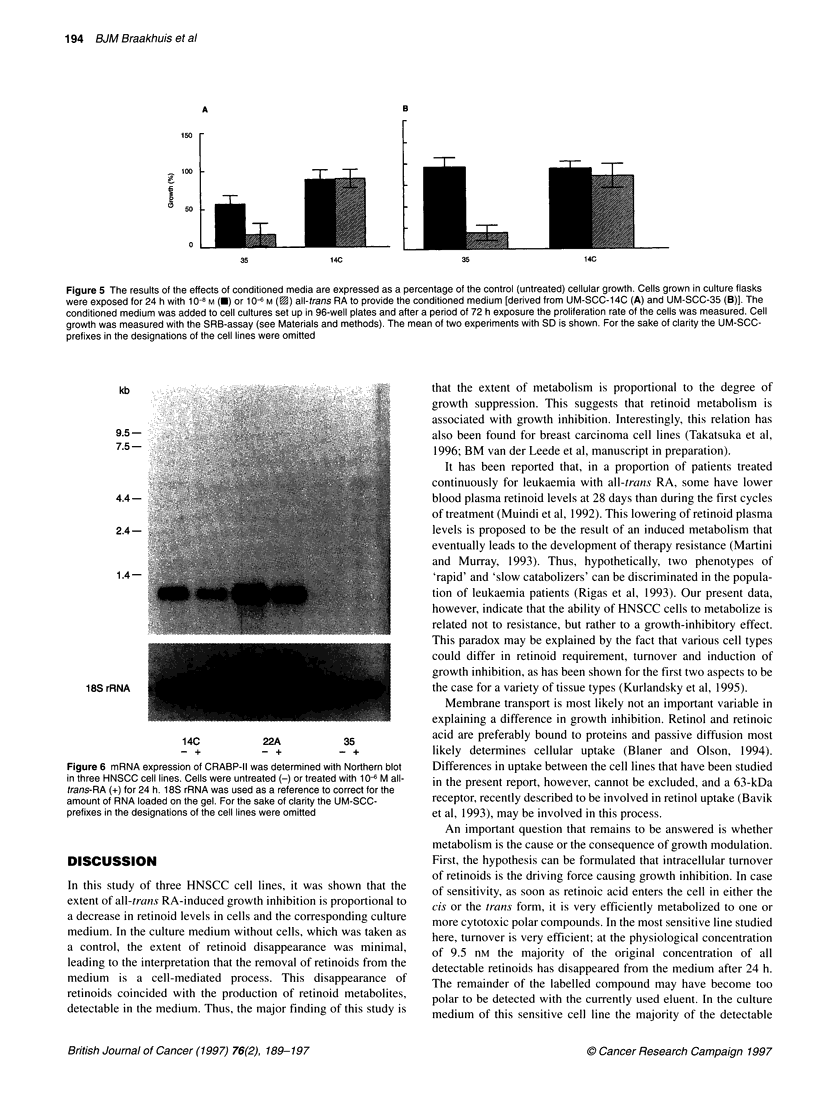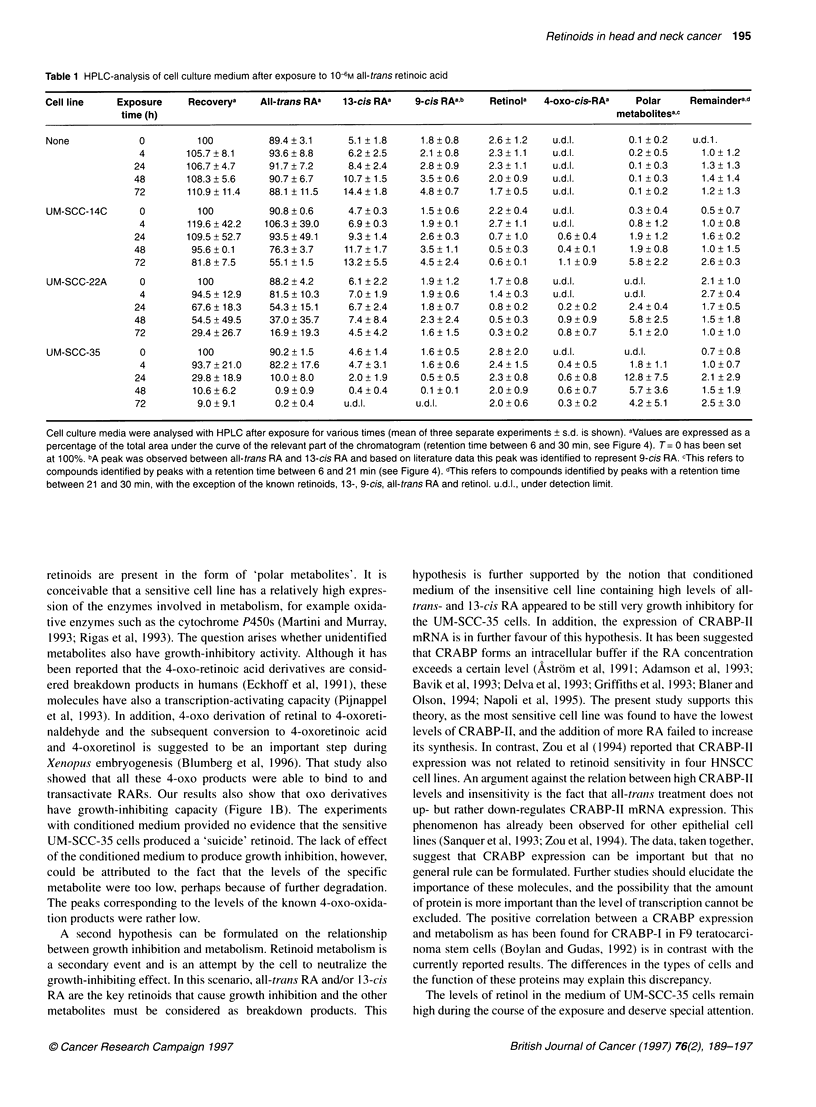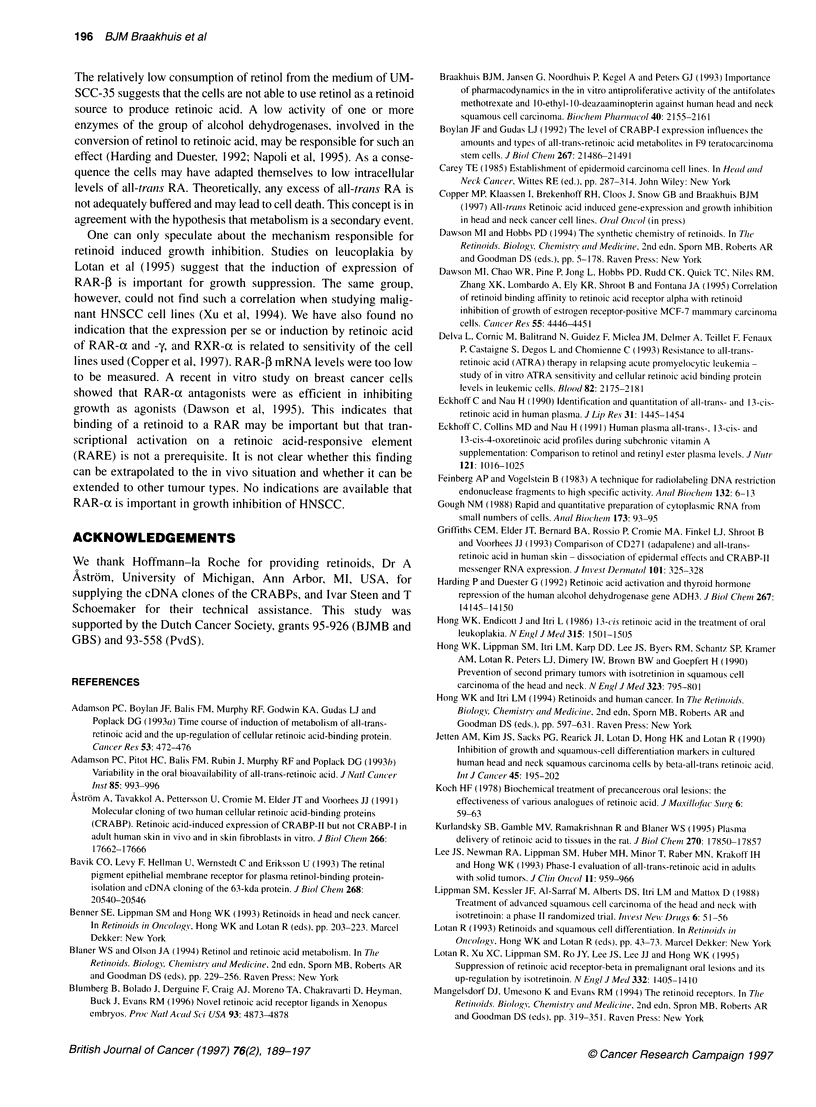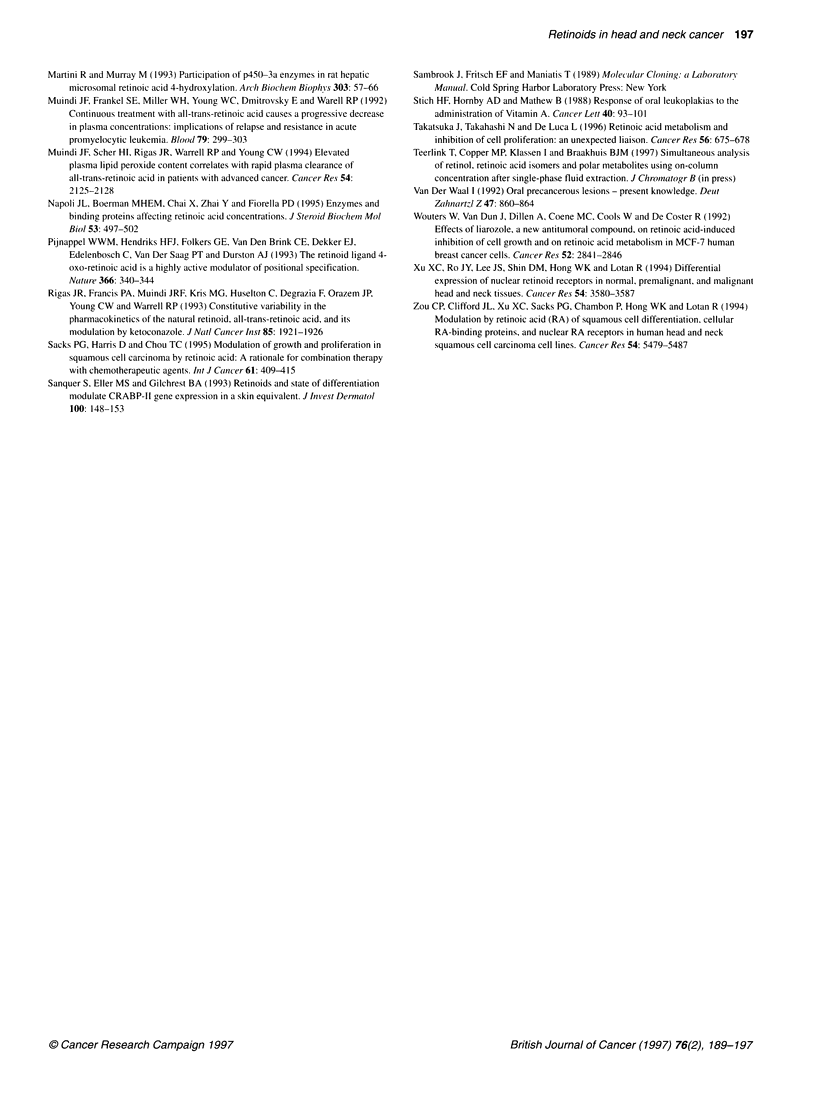# Retinoid metabolism and all-trans retinoic acid-induced growth inhibition in head and neck squamous cell carcinoma cell lines.

**DOI:** 10.1038/bjc.1997.361

**Published:** 1997

**Authors:** B. J. Braakhuis, I. Klaassen, B. M. van der Leede, J. Cloos, R. H. Brakenhoff, M. P. Copper, T. Teerlink, H. F. Hendriks, P. T. van der Saag, G. B. Snow

**Affiliations:** Department of Otolaryngology/Head and Neck Surgery, University Hospital Vrije Universiteit, Amsterdam, The Netherlands.

## Abstract

**Images:**


British Journal of Cancer (1997) 76(2), 189–197
© 1997 Cancer Research Campaign

### Retinoid metabolism and all-*trans* retinoic acid-induced growth inhibition in head and neck squamous cell carcinoma cell lines

**BJM Braakhuis[1], I Klaassen[1], BM van der Leede[2], J Cloos[1], RH Brakenhoff[1], MP Copper[1], T Teerlink[3], HFJ Hendriks[4], PT van der Saag[2] and GB Snow[1]**

[1]Department of Otolaryngology / Head and Neck Surgery, University Hospital Vrije Universiteit, Amsterdam; [2]Hubrecht Laboratory, Netherlands Institute for Developmental Biology, Utrecht; [3]Department of Clinical Chemistry, University Hospital Vrije Universieit, Amsterdam and [4]Department of Physiology and Kinetics, TNO Nutrition and Food Research Institute, Zeist, The Netherlands

**Summary** Retinoids can reverse potentially premalignant lesions and prevent second primary tumours in patients with head and neck squamous cell carcinoma (HNSCC). Furthermore, it has been reported that acquired resistance to all-*trans* retinoic acid (RA) in leukaemia is associated with decreased plasma peak levels, probably the result of enhanced retinoid metabolism. The aim of this study was to investigate the metabolism of retinoids and relate this to growth inhibition in HNSCC. Three HNSCC cell lines were selected on the basis of a large variation in the all-*trans* RA-induced growth inhibition. Cells were exposed to 9.5 nM (radioactive) for 4 and 24 h, and to 1 and 10 μM (non-radioactive) all-*trans* RA for 4, 24, 48 and 72 h, and medium and cells were analysed for retinoid metabolites. At all concentrations studied, the amount of growth inhibition was proportional to the extent at which all-*trans*-, 13- and 9-*cis* RA disappeared from the medium as well as from the cells. This turnover process coincided with the formation of a group of as yet unidentified polar retinoid metabolites. The level of mRNA of cellular RA-binding protein II (CRABP-II), involved in retinoid homeostasis, was inversely proportional to growth inhibition. These findings indicate that for HNSCC retinoid metabolism may be associated with growth inhibition.

**Keywords:** CRABP-II, head and neck cancer, metabolism, retinoid, squamous

Retinoids are a class of compounds that consists of the natural vitamin A derivatives, such as retinol, retinal, retinoic acid (RA) and their various metabolic products, and the synthetic derivatives that are structurally related to these natural compounds (Dawson and Hobbs, 1994). Natural retinoids are important for normal epithelial cell differentiation. A low vitamin A (retinol) plasma level and a low dietary intake of retinoids have been proven to be risk factors in various carcinomas (Hong and Itri, 1994). Many studies report inhibiting effects of exogenous retinoids on the induction and progression of cancer in various tissues (Lotan, 1993). As for solid tumours, retinoids are particularly important for head and neck squamous cell carcinoma (HNSCC) (Benner et al, 1993). Three retinoids, 13-*cis* retinoic acid (13-*cis* RA) (Hong et al, 1986), retinyl palmitate (Stich et al, 1988) and all-*trans* retinoic acid (all-*trans* RA) (Koch, 1978), cause responses in 40–70% of patients with leucoplakia, the most common premalignant lesion of the mucosa of the oral cavity (Van der Waal, 1992). It has also been demonstrated that administration of 13-*cis* RA could successfully prevent and/or delay the occurrence of second primary tumours in the upper aerodigestive tract (Hong et al, 1990). In a chemotherapeutic approach, single-agent 13-*cis* RA has limited activity in advanced squamous cell carcinoma of the head and neck (Linnman et al, 1988). Thus far, retinoids appear to be active in early-stage HNSCC, but their utility is limited by the interpatient variability with respect to not only response, but also to side-effects. Another characteristic of treatment of HNSCC with retinoids is that discontinuation of treatment leads invariably to recurrence of the lesion (Hong and Itri, 1994).

*Received 1 May 1996*
*Revised 9 January 1997*
*Accepted 14 January 1997*

*Correspondence to:* BJM Braakhuis, Department of Otolaryngology/Head and Neck Surgery, University Hospital Vrije Universiteit, De Boelelaan 1117, PO Box 7057, 1007 MB Amsterdam, The Netherlands

It is not known how retinoids are actually able to regulate growth control. The association between vitamin A deficiency and the development of cancer suggests that the intracellular retinoid-dependent pathways play a role in cancer development. Most of the actions of retinoids are thought to result from changes in gene expression mediated by nuclear retinoic acid receptors (RARs) and retinoid X receptors (RXRs) (Mangelsdorf et al, 1994). Retinoids bind to these receptors, which act as transcription factors upon dimerization. The expression of one such RAR-β, as determined by in situ hybridization was found to be selectively absent in 31 of 52 leucoplakia cases and could be restored by treatment with 13-*cis* RA (Lotan et al, 1995). Restoration of expression is associated with a clinical response to 13-*cis* RA. However, pretreatment levels of RAR-β do not predict the clinical response (Lotan et al, 1995). Sixty-five per cent of clinical HNSCC samples show a lack of RAR-β expression, as judged by in situ hybridization (Xu et al, 1994). No association, however, was found in HNSCC cell lines between all-*trans* RA sensitivity and the expression of RARs, RXRs and the cellular retinoic acid-binding proteins (CRABP) (Zou et al, 1994).

All-*trans* RA induces complete remission in most patients with acute promyelocytic leukaemia. However, relapses are frequent and resistance to the drug is developing. This resistance is associated with unexpectedly low plasma levels of retinoids despite continued treatment (Muindi et al, 1994). The interindividual variation in retinoid pharmacokinetics is already known from other studies (Eckhoff et al, 1991; Adamson et al, 1993; Lee et al, 1993), and it is hypothesized that the variability in the pharmacokinetics of all-*trans* RA may result from differences in catabolic rates determined or influenced by genetic or environmental factors. Thus, a poor metabolism may be associated with a response, whereas enhanced retinoid metabolism is associated with acquired therapy resistance (Muindi et al, 1994). CRABP-II levels may be the cause of this resistance, as increased levels of this enzyme have been found in tumour cells from relapsed patients treated with all-*trans* RA (Delva et al, 1993). Also, the oxidative breakdown via the cytochrome *P*450 enzyme system is a possible explanation (Rigas et al, 1993). Two lines of evidence for the latter possibility have been provided: a tenfold increase in the 4-oxo-all-*trans* RA glucuronide has been found in the urine of relapsed patients (Muindi et al, 1994) and ketoconazole and liarozole, inhibitors of the cytochrome *P*450 system, are able to attenuate this catabolism (Wouters et al, 1992; Rigas et al, 1993).

In vitro, variation in growth inhibition after exposure of HNSCC to all-*trans* RA has been reported (Jetten et al, 1990; Sacks et al, 1995), and thus far this variation cannot be explained (Zou et al, 1994). The aim of this study was to investigate the presence or absence of a variation in retinoid metabolism between HNSCC cell lines and whether this is related to the degree of growth inhibition by all-*trans* RA.

### MATERIALS AND METHODS

### Cell lines

HNSCC cell lines were obtained from Dr TE Carey, University of Michigan, Ann Arbor, MI, USA, and are described elsewhere (Carey, 1985). UM-SCC-14C originated from a local recurrence of cancer of the floor of the mouth, UM-SCC-22A and -35 from hypopharyngeal tumours. Cells were cultured routinely in DMEM (Dulbecco's modified Eagle medium, ICN Biomedicals, Irvine, UK) with 5% fetal calf serum (FCS, Flow Laboratories) in 75-cm$^2$ flasks (Nunc, Roskilde, Denmark). Cellular doubling times were 26 h for UM-SCC-14C, 52 h for UM-SCC-35 and 34 h for the UM-SCC-22A cell line.

### Chemicals

All-*trans* RA was obtained from Acros Chimica (Geel, Belgium), 4-oxo-*trans*- and *cis*-RA were kind gifts of Hoffmann–la Roche, Basle, Switzerland; retinol and 13-*cis*-RA were obtained from Sigma (St Louis, MO, USA). All compounds were dissolved as a $10^{-2}$ M stock in dimethylsulphoxide (DMSO, JT Baker, Deventer, The Netherlands) and stored at –80°C. For each experiment freshly prepared solutions were made, the first ($10^{-3}$ M) being made with DMSO. Subsequent dilutions were prepared in cell culture medium. All handling with retinoids was performed in subdued light, tubes were wrapped in aluminium foil and oxidation was prevented by replacing the air by nitrogen.

### Cell growth inhibition studies

Effects on the growth of HNSCC cells were determined using the 'SRB assay'. Details of the assay, which measures the cellular protein content, reflecting the actual cell number, have been described previously (Braakhuis et al, 1993). In short, cells were plated at a concentration of 1500 (UM-SCC-14C), 2000 (UM-SCC-22A) and 3000 (UM-SCC-35) cells per well in 150 μl of DMEM and 5% FCS, and were allowed to attach and grow for 72 h (the 'lag phase'). After this phase, it was found that control (incubated only with culture medium) cell growth was logarithmic for a period up to 96 h. Consequently, all-*trans* RA was added in 50 μl of medium, resulting in a final concentration that varied between $10^{-5}$ and $10^{-9}$ M. Growth was assessed after 72 h (the 'log phase'), by staining the cellular protein with sulphorhodamine B (SRB, Sigma) and spectrophotometric measurement of the absorption at 540 nm with a microplate reader. $IC_{50}$ values were estimated based on the absorption values and defined as the concentration that corresponded to a reduction in growth of 50% compared with values for untreated control cells. When using the highest concentration of all-*trans* RA, a 1% DMSO solution was present in the cell culture medium. Control experiments showed that exposing the cells to this level of DMSO without all-*trans* RA leads to a growth inhibition of between 10% and 25%.

In a separate set of experiments the effect of conditioned medium was tested on the growth rate of UM-SCC-14C and -35 cells. For this purpose flasks containing near-confluent UM-SCC-35- and -14C-cells were exposed to $10^{-6}$ and $10^{-8}$ M all-*trans* RA for 24 h. These conditioned media were added to cells growing in 96-well plates that were about to start their log phase. The cells were exposed to this conditioned medium for another 72 h and the level of growth inhibition was determined by the 'SRB assay', as described.

### Exposure to radioactive all-*trans* RA

Near-confluent cultures, growing under normal conditions, were treated for 4 and 24 h with 9.5 nM [11,12-$^3$H] all-*trans* RA (Dupont NEN Research Products, Dordrecht, The Netherlands, sp. act. 52.1 Ci mmol$^{-1}$). After incubation the medium was removed and saved at –80°C. Cells were rinsed with phosphate-buffered saline (pH 7.4, PBS), scraped in 1 ml of PBS and collected by centrifugation. Cell pellets were stored at –80°C until extraction. Retinoids were extracted and analysed by reversed-phase high-performance liquid chromatography (HPLC) as described previously (Pijnappel et al, 1993). The following standards were included: 13- and 9-*cis*- and all-*trans* RA. The experiment was performed in duplicate.

### Exposure to non-radioactive all-*trans* RA

Cells were cultured to near confluence in 75-cm$^2$ flasks with 5 ml of medium (DMEM plus FCS). The cells were exposed to 10 and 1 μM all-*trans* RA. At each time point (0, 4, 24, 48 and 72 h) 500 μl of the supernatant was taken from a separate flask and stored at –80°C in the dark until analysis. For the analysis of the intracellular concentration of retinoids, the flasks were washed twice with fresh PBS (pH 7.4) and the cells were trypsinized. The number of living cells (determined with trypan blue) was calculated and cell pellets were washed twice with PBS and stored at –80°C in the dark.

Non-radioactive retinoids were determined by reversed-phase HPLC after extraction with acetonitrile (Teerlink et al, 1997). A Waters (Milford, MA, USA) HPLC system was used, consisting of a model 717 plus automatic sample injector, a model 616 gradient pump, a model 486 UV detector, and a temperature control module and column heater. Mobile phase was degassed online using a

© Cancer Research Campaign 1997

model DG2410 degasser from Uniflows (Tokyo, Japan). Millennium 2010 software from Waters was used for instrument control and data acquisition. Separation was performed on a Spherisorb ODS2 3-μm column (100 × 4.6 mm) from Phase Separations (Deeside, UK) maintained at 30°C. Composition of the mobile phases and the binary gradient used were as described by Eckhoff and Nau (1990). UV detection was performed at 340 nm and retinoids were identified using external standardization. We included the following standards: 4-oxo-*trans* RA, 4-oxo-*cis* RA, 13-*cis* RA, all-*trans* RA and retinol. As we also intended to measure the levels of unknown retinoid metabolites, the results were expressed as a percentage of the total area under the curve of the relevant part of the chromatogram (retention time between 6 and 30 min).

### Measurement of CRABP mRNA levels

Total RNA was isolated from cultured cells according to Gough (1988). Total RNA (20 μg) was loaded on a 1% agarose formaldehyde gel and electrophoresed in 3-(*N*-morpholine)-propane sulphonic acid (MOPS) buffer essentially as described by Sambrook et al (1989). The RNA was Northern blotted by capillary transfer in 10 × saline sodium citrate (SSC) (Sambrook et al, 1989) onto genescreen plus filters (Dupont NEN). The filter was baked for 2 h at 80°C, prehybridized in 7% sodium dodecyl sulphate (SDS), 0.5 M sodium phosphate buffer, 2 mM EDTA, pH 7.0, for 2 h at 65°C, and after addition of the denatured probe, hybridized at 65°C for 16 h. The probes were made by labelling the isolated 0.6-kb *XbaI*/*Bam*HI fragment containing human CRABP-I cDNA (Åström et al, 1991), the 1-kb *Eco*RI fragment containing human CRABP-II cDNA (Åström et al, 1991) and the 0.2-kb fragment containing part of 18S rRNA cDNA with [α-$^{32}$P]dCTP to a specific activity of approximately $10^9$ dpm $\mu g^{-1}$ by multiprimed elongation (Feinberg and Vogelstein, 1983). After hybridization the filters were washed twice with 2 × SSC, 0.2% SDS and twice with 0.2 × SSC, 0.2% SDS, at 65°C for 15 min, and the bands visualized by autoradiography with Kodak X-AR 5 film using intensifying screens. 18S rRNA was used as an internal standard to correct for the amount of RNA loaded on the gel.

**Figure 1** The SRB test was used to assess the antiproliferative effect of all-*trans* RA in three HNSCC cell lines (**A**). □, UM-SCC-14C; ○, UM-SCC-22A; ▲, UM-SCC-35. The growth inhibiting effect of 4-*trans*-oxo RA on two HNSCC cell lines is shown in **B**. Results of three separate experiments are indicated (means ± s.d.)

### RESULTS

### Inhibition of cell proliferation

The three HNSCC cell lines were selected for their considerable difference in their response to all-*trans* RA (Figure 1A). UM-SCC-35 was the most sensitive line with an $IC_{50}$ value of 6.8 nM. UM-SCC-14C showed hardly any response, even after exposure to the relatively high concentration of $10^{-5}$ M. The third line, UM-SCC-22A, showed an intermediate type of response, with a moderate growth inhibition at the broad concentration range from $10^{-5}$ to $10^{-9}$ M.

### Metabolism of 9.5 nM[$^3$H]all-*trans* RA

The fate of [$^3$H]all-*trans* RA was studied in the media and the cell pellets of all three cell lines. The cells were exposed to 9.5 nM all-*trans* RA, a concentration that is in the range found in human plasma (Eckhoff et al, 1991). The concentration of [$^3$H]all-*trans* RA decreased in the medium and the time dependency of this effect differed between the cell lines (Figure 2). In UM-SCC-35 this decrease started at 4 h and led to a total loss at 24 h exposure (Figure 2G and H). A similar decrease was seen for 9- and 13-*cis* RA. For this cell line the contribution of all-*trans* RA to the total amount of retinoids was relatively low, being 22% at 4 h and 1.4% at 24 h. For the insensitive line, UM-SCC-14C, the concentration of all-*trans* RA in the medium was the highest (19.5%) of all three cell lines at 24 h (Figure 2C and D) and for the UM-SCC-22A a somewhat lower value (9.4%) was observed (Figure 2E and F). The HPLC method of analysis enabled us to measure retinoid metabolites. In the media of the cell cultures a number of peaks could be detected after 4 and 24 h exposure to [$^3$H]all-*trans* RA, corresponding to retention times between 2 and 20 min (Figure 2). For UM-SCC-35 these peaks formed at 24 h the majority (86 %) of the total of labelled retinoids. These polar metabolites were less prevalent in the two other cell lines, being 49% and 68% for UM-SCC-14 and 22A respectively.

As the intracellular recovery was rather low, varying between 0.2% and 2.5% of the total amount of radioactivity added, only estimations of retinoid levels could be made. Intracellular retinoid levels decreased in the course of time. UM-SCC-35 had the lowest

© Cancer Research Campaign 1997

**Figure 2** High-performance liquid chromatogram after 4 (left) and 24 h (right) exposure to 9.5 nM [$^3$H]all-*trans* RA. Results are expressed in c.p.m. on the *y*-axis after multiplication by $10^{-3}$. Note the difference in disappearance of retinoids and formation of metabolites between the cell lines. Data are shown for the culture media. We included the following standards with the corresponding retention times in minutes: 13-*cis*-RA (24.3), 9-*cis*-RA (25.5), all-*trans*-RA (26.2). (**A** and **B**) Medium without cells; (**C** and **D**) UM-SCC-14C; (**E** and **F**) UM-SCC-22A; and (**G** and **H**) UM-SCC-35. This experiment was performed in duplicate. A representative experiment is shown levels of intracellular retinoids, most of them being polar metabolites. The differences between the lines, however, were not as large as seen in the media.

The observed decrease in retinoid levels in the cell culture media is mainly due to retinoid turnover by the cells. Without cells the decrease in retinoid levels in the medium was minimal (Figure 2 A and B).

### Metabolism of 1 and 10 μM unlabelled all-*trans* RA

When exposed to 1 μM all-*trans* RA, retinoid metabolites were measured after various time points (Figure 3 and Table 1). In general, the pattern is similar to that seen with the exposure to the lower concentration, but the effect was less dramatic. Because now the exposure time is longer, the kinetics of disappearance of

© Cancer Research Campaign 1997

**Figure 3** Measurement of retinoids in cell culture media after various periods of exposure to $10^{-6}$ M all-*trans* RA. (**A**) values are expressed as a percentage of the total area under the curve of the relevant part of the chromatogram (retention time between 6 and 30 min, see Figure 4); *T* = 0 has been set at 100%. **B**, values are expressed as a percentage of the total area under the curve of the total chromatogram at that given time point

**Figure 4** HPLC chromatogram of the cell culture medium of UM-SCC-35, 24 h after exposure to $10^{-6}$ M all-*trans* RA. Peaks: 1, all-*trans* RA; 2, 9-*cis* RA; 3, 13-*cis* RA; 4, retinol; 5, 4-oxo-13-*cis* RA. Note the unidentified peaks between 6 and 21 min. AU, arbitrary units the retinoids can be studied in more detail. The disappearance of the major retinoids from the medium was highest for the UM-SCC-35 cell line. After 24 h this is already very significant and reaches an apparent plateau at 48 h with 10% of the original level. The disappearance of retinoids from the other two cell lines is more gradual, UM-SCC-14C being the slowest (Figure 3). With regard to the composition of the retinoids a shift could be observed: the contribution of the unidentifiable polar metabolites, visible as a number of peaks with retention times between 6 and 21 min, actually increased. These metabolites could be detected for all three cell lines, but was most remarkable for UM-SCC-35, followed in order by UM-SCC-22A and -14C. An example of high-performance liquid chromatogram of UM-SCC-35 after 24 h exposure is given in Figure 4.

After exposure to 1 μM all-*trans* RA, UM-SCC-35 had the lowest and UM-SCC-22A the highest levels of intracellular retinoids. The intracellular concentrations were too low to be considered accurate. Generally speaking, retinoid levels decreased in the course of time, but no polar metabolites could be detected.

When the cells of the three cell lines were exposed to 10 μM all-*trans* RA, the pattern seen was similar to that with the 1 μM exposure experiments (data not shown).

### Growth inhibition by retinoid metabolites

We wished to investigate whether growth inhibition in the UM-SCC-35 cell line could be caused by the excessive formation of one or more toxic retinoid metabolites. To test this hypothesis we performed two types of experiments. Two cell lines were exposed to a well-known polar metabolite of all-*trans* RA, 4-oxo-*trans* RA. This compound was found to induce growth inhibition in the UM-SCC-35 cell line (Figure 1B), although to a lesser extent than all-*trans* RA, $IC_{50}$ values being 39.0 and 6.8 nM respectively (Figure 1A). This metabolite was not active in the UM-SCC-14C cell line.

In a second set of experiments we argued that UM-SCC-35 cells might produce toxic metabolites and that these were released into the medium. Thus, UM-SCC-35 cells were exposed for 24 h to $10^{-6}$ and $10^{-8}$ M all-*trans* RA and it was found that this medium, when added to virgin UM-SCC-14C cells, minimally affected growth of these cells (Figure 4). Incubation of UM-SCC-35 cells with conditioned medium of UM-SCC-14C cells (after treatment with $10^{-8}$ M), however, caused a stronger antiproliferative effect than incubation with the medium derived from UM-SCC-35 cell cultures. It appears that newly formed retinoids are less potent with respect to growth inhibition than the parent compounds they were derived from, but it must be added that, on an individual basis, the concentration of these metabolites is significantly lower than the concentration of parent retinoid (Table 1).

### Measurement of CRABP expression

CRABP-I and -II are proteins involved in retinoid homeostasis and they may be important with respect to metabolism and growth inhibition. We therefore analysed CRABP-I and -II expression by Northern blotting and hybridization. CRABP-I had undetectable transcript levels in all cell lines, confirming previous results (Zou et al, 1994). The analysis of CRABP-II showed that UM-SCC-35 had considerably lower transcript levels than the other two cell lines (Figure 5). Exposure to $10^{-6}$ M all-*trans* RA for 24 h had an apparent down-regulating effect on CRABP-II mRNA levels in UM-SCC-14C and -22A.

**Figure 5** The results of the effects of conditioned media are expressed as a percentage of the control (untreated) cellular growth. Cells grown in culture flasks were exposed for 24 h with $10^{-8}$ M (■) or $10^{-6}$ M (▨) all-*trans* RA to provide the conditioned medium [derived from UM-SCC-14C (**A**) and UM-SCC-35 (**B**)]. The conditioned medium was added to cell cultures set up in 96-well plates and after a period of 72 h exposure the proliferation rate of the cells was measured. Cell growth was measured with the SRB-assay (see Materials and methods). The mean of two experiments with SD is shown. For the sake of clarity the UM-SCC-prefixes in the designations of the cell lines were omitted

**Figure 6** mRNA expression of CRABP-II was determined with Northern blot in three HNSCC cell lines. Cells were untreated (–) or treated with $10^{-6}$ M all-*trans*-RA (+) for 24 h. 18S rRNA was used as a reference to correct for the amount of RNA loaded on the gel. For the sake of clarity the UM-SCC-prefixes in the designations of the cell lines were omitted

### DISCUSSION

In this study of three HNSCC cell lines, it was shown that the extent of all-*trans* RA-induced growth inhibition is proportional to a decrease in retinoid levels in cells and the corresponding culture medium. In the culture medium without cells, which was taken as a control, the extent of retinoid disappearance was minimal, leading to the interpretation that the removal of retinoids from the medium is a cell-mediated process. This disappearance of retinoids coincided with the production of retinoid metabolites, detectable in the medium. Thus, the major finding of this study is that the extent of metabolism is proportional to the degree of growth suppression. This suggests that retinoid metabolism is associated with growth inhibition. Interestingly, this relation has also been found for breast carcinoma cell lines (Takatsuka et al, 1996; BM van der Leede et al, manuscript in preparation).

It has been reported that, in a proportion of patients treated continuously for leukaemia with all-*trans* RA, some have lower blood plasma retinoid levels at 28 days than during the first cycles of treatment (Muindi et al, 1992). This lowering of retinoid plasma levels is proposed to be the result of an induced metabolism that eventually leads to the development of therapy resistance (Martini and Murray, 1993). Thus, hypothetically, two phenotypes of 'rapid' and 'slow catabolizers' can be discriminated in the population of leukaemia patients (Rigas et al, 1993). Our present data, however, indicate that the ability of HNSCC cells to metabolize is related not to resistance, but rather to a growth-inhibitory effect. This paradox may be explained by the fact that various cell types could differ in retinoid requirement, turnover and induction of growth inhibition, as has been shown for the first two aspects to be the case for a variety of tissue types (Kurlandsky et al, 1995).

Membrane transport is most likely not an important variable in explaining a difference in growth inhibition. Retinol and retinoic acid are preferably bound to proteins and passive diffusion most likely determines cellular uptake (Blaner and Olson, 1994). Differences in uptake between the cell lines that have been studied in the present report, however, cannot be excluded, and a 63-kDa receptor, recently described to be involved in retinol uptake (Bavik et al, 1993), may be involved in this process.

An important question that remains to be answered is whether metabolism is the cause or the consequence of growth modulation. First, the hypothesis can be formulated that intracellular turnover of retinoids is the driving force causing growth inhibition. In case of sensitivity, as soon as retinoic acid enters the cell in either the *cis* or the *trans* form, it is very efficiently metabolized to one or more cytotoxic polar compounds. In the most sensitive line studied here, turnover is very efficient; at the physiological concentration of 9.5 nM the majority of the original concentration of all detectable retinoids has disappeared from the medium after 24 h. The remainder of the labelled compound may have become too polar to be detected with the currently used eluent. In the culture medium of this sensitive cell line the majority of the detectable

 © Cancer Research Campaign 1997

**Table 1** HPLC-analysis of cell culture medium after exposure to $10^{-6}$M all-*trans* retinoic acid

| Cell line | Exposure time (h) | Recovery[a] | All-*trans* RA[a] | 13-*cis* RA[a] | 9-*cis* RA[a,b] | Retinol[a] | 4-oxo-*cis*-RA[a] | Polar metabolites[a,c] | Remainder[a,d] |
|---|---|---|---|---|---|---|---|---|---|
| None | 0 | 100 | 89.4 ± 3.1 | 5.1 ± 1.8 | 1.8 ± 0.8 | 2.6 ± 1.2 | u.d.l. | 0.1 ± 0.2 | u.d.1. |
| | 4 | 105.7 ± 8.1 | 93.6 ± 8.8 | 6.2 ± 2.5 | 2.1 ± 0.8 | 2.3 ± 1.1 | u.d.l. | 0.2 ± 0.5 | 1.0 ± 1.2 |
| | 24 | 106.7 ± 4.7 | 91.7 ± 7.2 | 8.4 ± 2.4 | 2.8 ± 0.9 | 2.3 ± 1.1 | u.d.l. | 0.1 ± 0.3 | 1.3 ± 1.3 |
| | 48 | 108.3 ± 5.6 | 90.7 ± 6.7 | 10.7 ± 1.5 | 3.5 ± 0.6 | 2.0 ± 0.9 | u.d.l. | 0.1 ± 0.3 | 1.4 ± 1.4 |
| | 72 | 110.9 ± 11.4 | 88.1 ± 11.5 | 14.4 ± 1.8 | 4.8 ± 0.7 | 1.7 ± 0.5 | u.d.l. | 0.1 ± 0.2 | 1.2 ± 1.3 |
| UM-SCC-14C | 0 | 100 | 90.8 ± 0.6 | 4.7 ± 0.3 | 1.5 ± 0.6 | 2.2 ± 0.4 | u.d.l. | 0.3 ± 0.4 | 0.5 ± 0.7 |
| | 4 | 119.6 ± 42.2 | 106.3 ± 39.0 | 6.9 ± 0.3 | 1.9 ± 0.1 | 2.7 ± 1.1 | u.d.l. | 0.8 ± 1.2 | 1.0 ± 0.8 |
| | 24 | 109.5 ± 52.7 | 93.5 ± 49.1 | 9.3 ± 1.4 | 2.6 ± 0.3 | 0.7 ± 1.0 | 0.6 ± 0.4 | 1.9 ± 1.2 | 1.6 ± 0.2 |
| | 48 | 95.6 ± 0.1 | 76.3 ± 3.7 | 11.7 ± 1.7 | 3.5 ± 1.1 | 0.5 ± 0.3 | 0.4 ± 0.1 | 1.9 ± 0.8 | 1.0 ± 1.5 |
| | 72 | 81.8 ± 7.5 | 55.1 ± 1.5 | 13.2 ± 5.5 | 4.5 ± 2.4 | 0.6 ± 0.1 | 1.1 ± 0.9 | 5.8 ± 2.2 | 2.6 ± 0.3 |
| UM-SCC-22A | 0 | 100 | 88.2 ± 4.2 | 6.1 ± 2.2 | 1.9 ± 1.2 | 1.7 ± 0.8 | u.d.l. | u.d.l. | 2.1 ± 1.0 |
| | 4 | 94.5 ± 12.9 | 81.5 ± 10.3 | 7.0 ± 1.9 | 1.9 ± 0.6 | 1.4 ± 0.3 | u.d.l. | u.d.l. | 2.7 ± 0.4 |
| | 24 | 67.6 ± 18.3 | 54.3 ± 15.1 | 6.7 ± 2.4 | 1.8 ± 0.7 | 0.8 ± 0.2 | 0.2 ± 0.2 | 2.4 ± 0.4 | 1.7 ± 0.5 |
| | 48 | 54.5 ± 49.5 | 37.0 ± 35.7 | 7.4 ± 8.4 | 2.3 ± 2.4 | 0.5 ± 0.3 | 0.9 ± 0.9 | 5.8 ± 2.5 | 1.5 ± 1.8 |
| | 72 | 29.4 ± 26.7 | 16.9 ± 19.3 | 4.5 ± 4.2 | 1.6 ± 1.5 | 0.3 ± 0.2 | 0.8 ± 0.7 | 5.1 ± 2.0 | 1.0 ± 1.0 |
| UM-SCC-35 | 0 | 100 | 90.2 ± 1.5 | 4.6 ± 1.4 | 1.6 ± 0.5 | 2.8 ± 2.0 | u.d.l. | u.d.l. | 0.7 ± 0.8 |
| | 4 | 93.7 ± 21.0 | 82.2 ± 17.6 | 4.7 ± 3.1 | 1.6 ± 0.6 | 2.4 ± 1.5 | 0.4 ± 0.5 | 1.8 ± 1.1 | 1.0 ± 0.7 |
| | 24 | 29.8 ± 18.9 | 10.0 ± 8.0 | 2.0 ± 1.9 | 0.5 ± 0.5 | 2.3 ± 0.8 | 0.6 ± 0.8 | 12.8 ± 7.5 | 2.1 ± 2.9 |
| | 48 | 10.6 ± 6.2 | 0.9 ± 0.9 | 0.4 ± 0.4 | 0.1 ± 0.1 | 2.0 ± 0.9 | 0.6 ± 0.7 | 5.7 ± 3.6 | 1.5 ± 1.9 |
| | 72 | 9.0 ± 9.1 | 0.2 ± 0.4 | u.d.l. | u.d.l. | 2.0 ± 0.6 | 0.3 ± 0.2 | 4.2 ± 5.1 | 2.5 ± 3.0 |

Cell culture media were analysed with HPLC after exposure for various times (mean of three separate experiments ± s.d. is shown). [a]Values are expressed as a percentage of the total area under the curve of the relevant part of the chromatogram (retention time between 6 and 30 min, see Figure 4). $T = 0$ has been set at 100%. [b]A peak was observed between all-*trans* RA and 13-*cis* RA and based on literature data this peak was identified to represent 9-*cis* RA. [c]This refers to compounds identified by peaks with a retention time between 6 and 21 min (see Figure 4). [d]This refers to compounds identified by peaks with a retention time between 21 and 30 min, with the exception of the known retinoids, 13-, 9-*cis*, all-*trans* RA and retinol. u.d.l., under detection limit.

retinoids are present in the form of 'polar metabolites'. It is conceivable that a sensitive cell line has a relatively high expression of the enzymes involved in metabolism, for example oxidative enzymes such as the cytochrome *P*450s (Martini and Murray, 1993; Rigas et al, 1993). The question arises whether unidentified metabolites also have growth-inhibitory activity. Although it has been reported that the 4-oxo-retinoic acid derivatives are considered breakdown products in humans (Eckhoff et al, 1991), these molecules have also a transcription-activating capacity (Pijnappel et al, 1993). In addition, 4-oxo derivation of retinal to 4-oxoretinaldehyde and the subsequent conversion to 4-oxoretinoic acid and 4-oxoretinol is suggested to be an important step during *Xenopus* embryogenesis (Blumberg et al, 1996). That study also showed that all these 4-oxo products were able to bind to and transactivate RARs. Our results also show that oxo derivatives have growth-inhibiting capacity (Figure 1B). The experiments with conditioned medium provided no evidence that the sensitive UM-SCC-35 cells produced a 'suicide' retinoid. The lack of effect of the conditioned medium to produce growth inhibition, however, could be attributed to the fact that the levels of the specific metabolite were too low, perhaps because of further degradation. The peaks corresponding to the levels of the known 4-oxo-oxidation products were rather low.

A second hypothesis can be formulated on the relationship between growth inhibition and metabolism. Retinoid metabolism is a secondary event and is an attempt by the cell to neutralize the growth-inhibiting effect. In this scenario, all-*trans* RA and/or 13-*cis* RA are the key retinoids that cause growth inhibition and the other metabolites must be considered as breakdown products. This hypothesis is further supported by the notion that conditioned medium of the insensitive cell line containing high levels of all-*trans*- and 13-*cis* RA appeared to be still very growth inhibitory for the UM-SCC-35 cells. In addition, the expression of CRABP-II mRNA is in further favour of this hypothesis. It has been suggested that CRABP forms an intracellular buffer if the RA concentration exceeds a certain level (Åström et al, 1991; Adamson et al, 1993; Bavik et al, 1993; Delva et al, 1993; Griffiths et al, 1993; Blaner and Olson, 1994; Napoli et al, 1995). The present study supports this theory, as the most sensitive cell line was found to have the lowest levels of CRABP-II, and the addition of more RA failed to increase its synthesis. In contrast, Zou et al (1994) reported that CRABP-II expression was not related to retinoid sensitivity in four HNSCC cell lines. An argument against the relation between high CRABP-II levels and insensitivity is the fact that all-*trans* treatment does not up- but rather down-regulates CRABP-II mRNA expression. This phenomenon has already been observed for other epithelial cell lines (Sanquer et al, 1993; Zou et al, 1994). The data, taken together, suggest that CRABP expression can be important but that no general rule can be formulated. Further studies should elucidate the importance of these molecules, and the possibility that the amount of protein is more important than the level of transcription cannot be excluded. The positive correlation between a CRABP expression and metabolism as has been found for CRABP-I in F9 teratocarcinoma stem cells (Boylan and Gudas, 1992) is in contrast with the currently reported results. The differences in the types of cells and the function of these proteins may explain this discrepancy.

The levels of retinol in the medium of UM-SCC-35 cells remain high during the course of the exposure and deserve special attention.

© Cancer Research Campaign 1997

The relatively low consumption of retinol from the medium of UM-SCC-35 suggests that the cells are not able to use retinol as a retinoid source to produce retinoic acid. A low activity of one or more enzymes of the group of alcohol dehydrogenases, involved in the conversion of retinol to retinoic acid, may be responsible for such an effect (Harding and Duester, 1992; Napoli et al, 1995). As a consequence the cells may have adapted themselves to low intracellular levels of all-*trans* RA. Theoretically, any excess of all-*trans* RA is not adequately buffered and may lead to cell death. This concept is in agreement with the hypothesis that metabolism is a secondary event.

One can only speculate about the mechanism responsible for retinoid induced growth inhibition. Studies on leucoplakia by Lotan et al (1995) suggest that the induction of expression of RAR-β is important for growth suppression. The same group, however, could not find such a correlation when studying malignant HNSCC cell lines (Xu et al, 1994). We have also found no indication that the expression per se or induction by retinoic acid of RAR-α and -γ, and RXR-α is related to sensitivity of the cell lines used (Copper et al, 1997). RAR-β mRNA levels were too low to be measured. A recent in vitro study on breast cancer cells showed that RAR-α antagonists were as efficient in inhibiting growth as agonists (Dawson et al, 1995). This indicates that binding of a retinoid to a RAR may be important but that transcriptional activation on a retinoic acid-responsive element (RARE) is not a prerequisite. It is not clear whether this finding can be extrapolated to the in vivo situation and whether it can be extended to other tumour types. No indications are available that RAR-α is important in growth inhibition of HNSCC.

### ACKNOWLEDGEMENTS

We thank Hoffmann–la Roche for providing retinoids, Dr A Åström, University of Michigan, Ann Arbor, MI, USA, for supplying the cDNA clones of the CRABPs, and Ivar Steen and T Schoemaker for their technical assistance. This study was supported by the Dutch Cancer Society, grants 95-926 (BJMB and GBS) and 93-558 (PvdS).

### REFERENCES

Adamson PC, Boylan JF, Balis FM, Murphy RF, Godwin KA, Gudas LJ and Poplack DG (1993*a*) Time course of induction of metabolism of all-trans-retinoic acid and the up-regulation of cellular retinoic acid-binding protein. *Cancer Res* **53**: 472–476

Adamson PC, Pitot HC, Balis FM, Rubin J, Murphy RF and Poplack DG (1993*b*) Variability in the oral bioavailability of all-trans-retinoic acid. *J Natl Cancer Inst* **85**: 993–996

Åström A, Tavakkol A, Pettersson U, Cromie M, Elder JT and Voorhees JJ (1991) Molecular cloning of two human cellular retinoic acid-binding proteins (CRABP). Retinoic acid-induced expression of CRABP-II but not CRABP-I in adult human skin in vivo and in skin fibroblasts in vitro. *J Biol Chem* **266**: 17662–17666

Bavik CO, Levy F, Hellman U, Wernstedt C and Eriksson U (1993) The retinal pigment epithelial membrane receptor for plasma retinol-binding protein-isolation and cDNA cloning of the 63-kda protein. *J Biol Chem* **268**: 20540–20546

Benner SE, Lippman SM and Hong WK (1993) Retinoids in head and neck cancer. In *Retinoids in Oncology*, Hong WK and Lotan R (eds), pp. 203–223. Marcel Dekker: New York

Blaner WS and Olson JA (1994) Retinol and retinoic acid metabolism. In *The Retinoids. Biology, Chemistry and Medicine*, 2nd edn, Sporn MB, Roberts AR and Goodman DS (eds), pp. 229–256. Raven Press: New York

Blumberg B, Bolado J, Derguine F, Craig AJ, Moreno TA, Chakravarti D, Heyman, Buck J, Evans RM (1996) Novel retinoic acid receptor ligands in Xenopus embryos. *Proc Natl Acad Sci USA* **93**: 4873–4878

Braakhuis BJM, Jansen G, Noordhuis P, Kegel A and Peters GJ (1993) Importance of pharmacodynamics in the in vitro antiproliferative activity of the antifolates methotrexate and 10-ethyl-10-deazaaminopterin against human head and neck squamous cell carcinoma. *Biochem Pharmacol* **40**: 2155–2161

Boylan JF and Gudas LJ (1992) The level of CRABP-I expression influences the amounts and types of all-trans-retinoic acid metabolites in F9 teratocarcinoma stem cells. *J Biol Chem* **267**: 21486–21491

Carey TE (1985) Establishment of epidermoid carcinoma cell lines. In *Head and Neck Cancer*, Wittes RE (ed.), pp. 287–314. John Wiley: New York

Copper MP, Klaassen I, Brekenhoff RH, Cloos J, Snow GB and Braakhuis BJM (1997) All-*trans* Retinoic acid induced gene-expression and growth inhibition in head and neck cancer cell lines. *Oral Oncol* (in press)

Dawson MI and Hobbs PD (1994) The synthetic chemistry of retinoids. In *The Retinoids. Biology, Chemistry and Medicine*, 2nd edn, Sporn MB, Roberts AR and Goodman DS (eds.), pp. 5–178. Raven Press: New York

Dawson MI, Chao WR, Pine P, Jong L, Hobbs PD, Rudd CK, Quick TC, Niles RM, Zhang XK, Lombardo A, Ely KR, Shroot B and Fontana JA (1995) Correlation of retinoid binding affinity to retinoic acid receptor alpha with retinoid inhibition of growth of estrogen receptor-positive MCF-7 mammary carcinoma cells. *Cancer Res* **55**: 4446–4451

Delva L, Cornic M, Balitrand N, Guidez F, Miclea JM, Delmer A, Teillet F, Fenaux P, Castaigne S, Degos L and Chomienne C (1993) Resistance to all-trans-retinoic acid (ATRA) therapy in relapsing acute promyelocytic leukemia – study of in vitro ATRA sensitivity and cellular retinoic acid binding protein levels in leukemic cells. *Blood* **82**: 2175–2181

Eckhoff C and Nau H (1990) Identification and quantitation of all-trans- and 13-cis-retinoic acid in human plasma. *J Lip Res* **31**: 1445–1454

Eckhoff C, Collins MD and Nau H (1991) Human plasma all-trans-, 13-cis- and 13-cis-4-oxoretinoic acid profiles during subchronic vitamin A supplementation: Comparison to retinol and retinyl ester plasma levels. *J Nutr* **121**: 1016–1025

Feinberg AP and Vogelstein B (1983) A technique for radiolabeling DNA restriction endonuclease fragments to high specific activity. *Anal Biochem* **132**: 6–13

Gough NM (1988) Rapid and quantitative preparation of cytoplasmic RNA from small numbers of cells. *Anal Biochem* **173**: 93–95

Griffiths CEM, Elder JT, Bernard BA, Rossio P, Cromie MA, Finkel LJ, Shroot B and Voorhees JJ (1993) Comparison of CD271 (adapalene) and all-trans-retinoic acid in human skin – dissociation of epidermal effects and CRABP-II messenger RNA expression. *J Invest Dermatol* **101**: 325–328

Harding P and Duester G (1992) Retinoic acid activation and thyroid hormone repression of the human alcohol dehydrogenase gene ADH3. *J Biol Chem* **267**: 14145–14150

Hong WK, Endicott J and Itri L (1986) 13-*cis* retinoic acid in the treatment of oral leukoplakia. *N Engl J Med* **315**: 1501–1505

Hong WK, Lippman SM, Itri LM, Karp DD, Lee JS, Byers RM, Schantz SP, Kramer AM, Lotan R, Peters LJ, Dimery IW, Brown BW and Goepfert H (1990) Prevention of second primary tumors with isotretinion in squamous cell carcinoma of the head and neck. *N Engl J Med* **323**: 795–801

Hong WK and Itri LM (1994) Retinoids and human cancer. In *The Retinoids. Biology, Chemistry and Medicine*, 2nd edn, Sporn MB, Roberts AR and Goodman DS (eds.), pp. 597–631. Raven Press: New York

Jetten AM, Kim JS, Sacks PG, Rearick JI, Lotan D, Hong HK and Lotan R (1990) Inhibition of growth and squamous-cell differentiation markers in cultured human head and neck squamous carcinoma cells by beta-all-trans retinoic acid. *Int J Cancer* **45**: 195–202

Koch HF (1978) Biochemical treatment of precancerous oral lesions: the effectiveness of various analogues of retinoic acid. *J Maxillofac Surg* **6**: 59–63

Kurlandsky SB, Gamble MV, Ramakrishnan R and Blaner WS (1995) Plasma delivery of retinoic acid to tissues in the rat. *J Biol Chem* **270**: 17850–17857

Lee JS, Newman RA, Lippman SM, Huber MH, Minor T, Raber MN, Krakoff IH and Hong WK (1993) Phase-I evaluation of all-trans-retinoic acid in adults with solid tumors. *J Clin Oncol* **11**: 959–966

Lippman SM, Kessler JF, Al-Sarraf M, Alberts DS, Itri LM and Mattox D (1988) Treatment of advanced squamous cell carcinoma of the head and neck with isotretinoin: a phase II randomized trial. *Invest New Drugs* **6**: 51–56

Lotan R (1993) Retinoids and squamous cell differentiation. In *Retinoids in Oncology*, Hong WK and Lotan R (eds), pp. 43–73. Marcel Dekker: New York

Lotan R, Xu XC, Lippman SM, Ro JY, Lee JS, Lee JJ and Hong WK (1995) Suppression of retinoic acid receptor-beta in premalignant oral lesions and its up-regulation by isotretinoin. *N Engl J Med* **332**: 1405–1410

Mangelsdorf DJ, Umesono K and Evans RM (1994) The retinoid receptors. In *The Retinoids. Biology, Chemistry and Medicine*, 2nd edn, Spron MB, Roberts AR and Goodman DS (eds), pp. 319–351. Raven Press: New York

© Cancer Research Campaign 1997

Martini R and Murray M (1993) Participation of p450–3a enzymes in rat hepatic microsomal retinoic acid 4-hydroxylation. *Arch Biochem Biophys* **303**: 57–66

Muindi JF, Frankel SE, Miller WH, Young WC, Dmitrovsky E and Warell RP (1992) Continuous treatment with all-trans-retinoic acid causes a progressive decrease in plasma concentrations: implications of relapse and resistance in acute promyelocytic leukemia. *Blood* **79**: 299–303

Muindi JF, Scher HI, Rigas JR, Warrell RP and Young CW (1994) Elevated plasma lipid peroxide content correlates with rapid plasma clearance of all-trans-retinoic acid in patients with advanced cancer. *Cancer Res* **54**: 2125–2128

Napoli JL, Boerman MHEM, Chai X, Zhai Y and Fiorella PD (1995) Enzymes and binding proteins affecting retinoic acid concentrations. *J Steroid Biochem Mol Biol* **53**: 497–502

Pijnappel WWM, Hendriks HFJ, Folkers GE, Van Den Brink CE, Dekker EJ, Edelenbosch C, Van Der Saag PT and Durston AJ (1993) The retinoid ligand 4-oxo-retinoic acid is a highly active modulator of positional specification. *Nature* **366**: 340–344

Rigas JR, Francis PA, Muindi JRF, Kris MG, Huselton C, Degrazia F, Orazem JP, Young CW and Warrell RP (1993) Constitutive variability in the pharmacokinetics of the natural retinoid, all-trans-retinoic acid, and its modulation by ketoconazole. *J Natl Cancer Inst* **85**: 1921–1926

Sacks PG, Harris D and Chou TC (1995) Modulation of growth and proliferation in squamous cell carcinoma by retinoic acid: A rationale for combination therapy with chemotherapeutic agents. *Int J Cancer* **61**: 409–415

Sanquer S, Eller MS and Gilchrest BA (1993) Retinoids and state of differentiation modulate CRABP-II gene expression in a skin equivalent. *J Invest Dermatol* **100**: 148–153

Sambrook J, Fritsch EF and Maniatis T (1989) *Molecular Cloning: a Laboratory Manual*. Cold Spring Harbor Laboratory Press: New York

Stich HF, Hornby AD and Mathew B (1988) Response of oral leukoplakias to the administration of Vitamin A. *Cancer Lett* **40**: 93–101

Takatsuka J, Takahashi N and De Luca L (1996) Retinoic acid metabolism and inhibition of cell proliferation: an unexpected liaison. *Cancer Res* **56**: 675–678

Teerlink T, Copper MP, Klassen I and Braakhuis BJM (1997) Simultaneous analysis of retinol, retinoic acid isomers and polar metabolites using on-column concentration after single-phase fluid extraction. *J Chromatogr B* (in press)

Van Der Waal I (1992) Oral precancerous lesions – present knowledge. *Deut Zahnartzl Z* **47**: 860–864

Wouters W, Van Dun J, Dillen A, Coene MC, Cools W and De Coster R (1992) Effects of liarozole, a new antitumoral compound, on retinoic acid-induced inhibition of cell growth and on retinoic acid metabolism in MCF-7 human breast cancer cells. *Cancer Res* **52**: 2841–2846

Xu XC, Ro JY, Lee JS, Shin DM, Hong WK and Lotan R (1994) Differential expression of nuclear retinoid receptors in normal, premalignant, and malignant head and neck tissues. *Cancer Res* **54**: 3580–3587

Zou CP, Clifford JL, Xu XC, Sacks PG, Chambon P, Hong WK and Lotan R (1994) Modulation by retinoic acid (RA) of squamous cell differentiation, cellular RA-binding proteins, and nuclear RA receptors in human head and neck squamous cell carcinoma cell lines. *Cancer Res* **54**: 5479–5487

© Cancer Research Campaign 1997